# Case Report: DGAT1 deficiency in three infants including a novel missense variant: structural insights and comparison with reported cases

**DOI:** 10.3389/fped.2026.1829390

**Published:** 2026-05-12

**Authors:** Elie G. Malki, Ahmad Adawi, Mayar Idkedek, Yaqoub Ashhab, Nadirah Damseh, Mutaz Sultan

**Affiliations:** 1 Medical Research Club, Faculty of Medicine, Al-Quds University, Abu Deis, Palestine; 2 Palestine-Korea Biotechnology Center, Palestine Polytechnic University, Hebron, Palestine; 3 Department of Pediatrics, Al-Makassed Hospital, East Jerusalem, Palestine

**Keywords:** consanguinity, diacylglycerol O-acyltransferase, diarrhea, exome sequencing, protein-losing enteropathies

## Abstract

**Introduction:**

DGAT1 deficiency is a rare cause of congenital diarrhea and protein-losing enteropathy, with only 39 reported cases and limited characterization of missense variants. This study integrates molecular interpretation with clinical data to refine genotype–phenotype correlations.

**Case description:**

Three Palestinian infants with DGAT1 deficiency were analyzed, and a structured literature search was conducted. The diagnosis was established by whole-exome sequencing, with confirmatory Sanger sequencing. A novel variant underwent segregation testing, conservation assessment, and three-dimensional protein modeling using cryo-electron microscopy structures. All patients presented with congenital diarrhea, hypoalbuminemia, and severe growth faltering. Two known loss-of-function variants (c.1183C>T; p.Arg395Ter and c.895-1G>A) and one novel homozygous missense variant (c.820C>T; p.Arg274Trp) were identified. Structural modeling localized the residue to the substrate tunnel, where substitution of arginine with tryptophan is predicted to disrupt acyl-CoA entry into the catalytic chamber. Literature comparison suggests a partial residual function consistent with later presentation. All patients improved with low-fat, amino acid–based nutrition.

**Conclusion:**

These findings expand the DGAT1 mutational spectrum and support the potential value of structure-informed analysis in the interpretation of rare missense variants, while highlighting the importance of early recognition and targeted nutritional therapy.

## Introduction

Congenital Diarrhea and Enteropathies (CODE) are a diverse group of malabsorption syndromes presenting in the neonatal or infancy period with severe diarrhea and failure to thrive (1). CODEs are categorized into five mechanistic groups affecting epithelial transport, metabolism, polarity, enteroendocrine function, or immune regulation. A rare disorder within the category of diseases affecting epithelial enzymes and metabolism is Diarrhea 7, Protein-Losing Enteropathy Type [DIAR7; Online Mendelian Inheritance in Man (OMIM) 615863]. DIAR7 results from germline mutations impairing Diacylglycerol O-Acyltransferase 1 (DGAT1), a key lipid-metabolizing enzyme highly expressed in the intestines. DGAT1 is an endoplasmic reticulum enzyme catalyzing the final step of triacylglycerol synthesis (2).

To date, most *DGAT1* variants reported in DIAR7 are biallelic truncating loss-of-function alleles, with few pathogenic missense variants in ClinVar (accessed Dec 7, 2025). Most missense variants are hypomorphic and retain partial activity, except when they affect the catalytic chamber or substrate-entry gateways. This aligns with *DGAT1*'s weak missense constraint in Genome Aggregation Database (gnomAD) v4.1 (accessed Dec 7, 2025).

DIAR7 is an exceedingly rare genetic disorder, with 39 affected individuals reported in the literature to date. Here, we describe three patients of Palestinian ancestry who presented to Al-Makassed Hospital (Jerusalem) with a consistent phenotype, including a novel *DGAT1* variant. We aim to raise awareness and highlight key diagnostic and management considerations.

## Case description

### Clinical features

**Figure 1:**
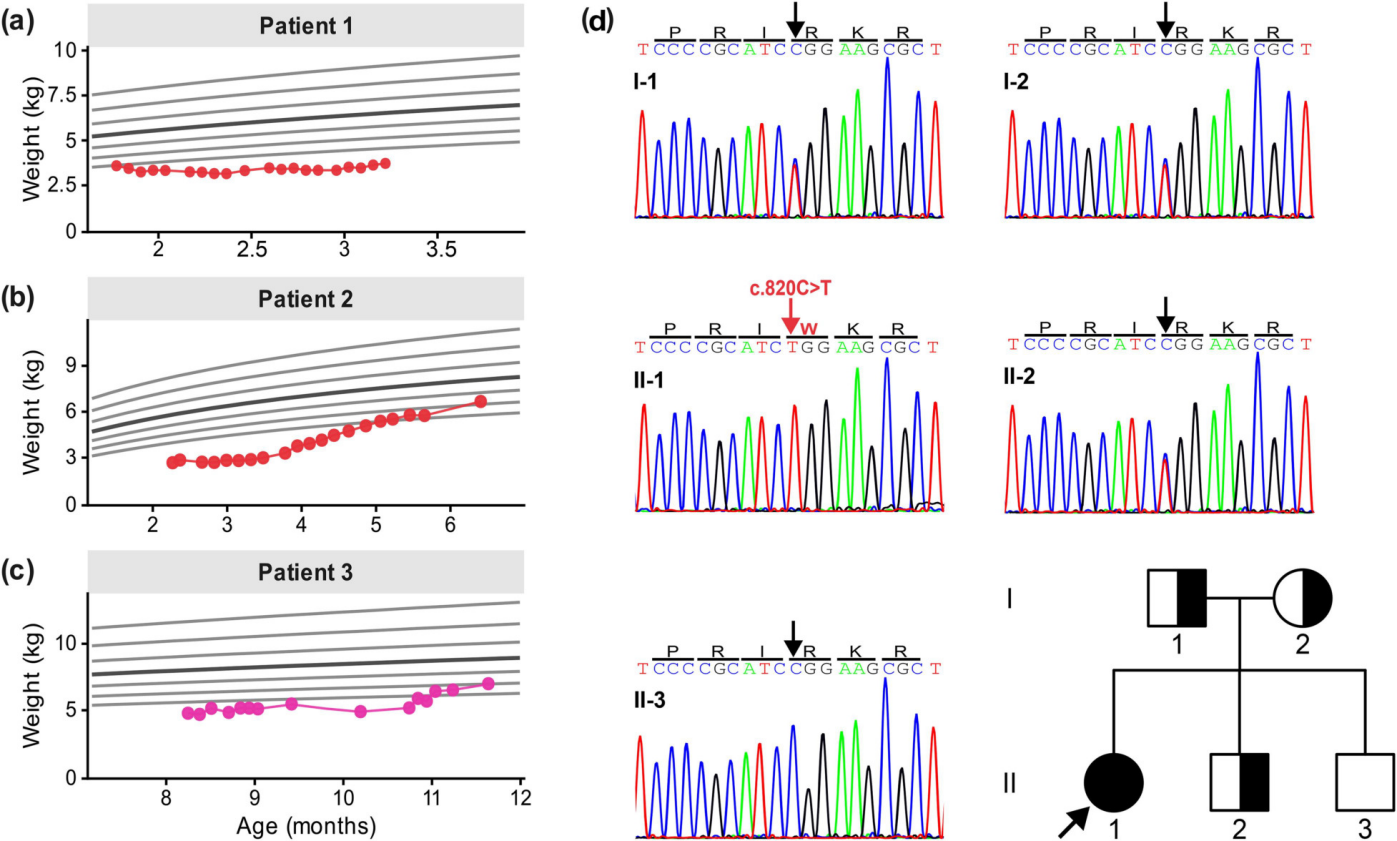
Weight-for-age trajectories of three infants with DGAT1 deficiency and segregation analysis of patient 3. Growth trajectories of Patient 1 **(a)**, Patient 2 **(b)**, and Patient 3 **(c)** plotted against the World Health Organization (WHO) weight-for-age reference curves. Red points correspond to individual weight measurements. Light gray lines represent the standard deviation bands from Z = −3 to Z = 3, and the dark gray line corresponds to the mean. **(d)** Sanger sequencing electropherograms demonstrating variant segregation in the family of Patient 3 with a two-generation pedigree. The proband (II-1, arrow) is homozygous for the DGAT1 c.820C>T missense variant. Both parents (I-1 and I-2) and one male sibling (II-2) are heterozygous carriers and clinically unaffected. The remaining sibling (II-3) displays a homozygous wildtype genotype.

Patient 1 (P1) was a full-term male neonate, born via spontaneous vaginal delivery with a birth weight of 3.2 kg. He was born to a consanguineous couple. By day 10 of life, while receiving mixed feeds, he developed profuse watery diarrhea. At presentation to our facility at 40 days of life, he appeared markedly cachectic and weighed 3.1 kg (Z-score −2.5 according to WHO anthropometric growth charts; Figure 1a). Physical examination showed abdominal distension and severe diaper rash.

Endoscopic evaluation showed standard villous architecture, and histopathology revealed no inflammatory infiltrates. The immunodeficiency workup revealed decreased serum immunoglobulin quantification with normal T-cell subset analysis. Nutritional management included trials of hydrolyzed and amino acid–based formulas, medium-chain triglyceride (MCT) oil supplementation, and intravenous immunoglobulin (IVIG) for hypogammaglobulinemia. Despite these interventions, growth remained severely impaired, with weight increasing to 3.7 kg (Z-score: −4.4) at 3 months of age. Whole exome sequencing (WES) confirmed the diagnosis of DGAT1 deficiency. The patient was last seen at 3 months of age, and he was lost to follow-up. Despite multiple attempts to contact the family, we were informed that he is continuing his care at a local hospital; however, no further clinical information could be attained.

Patient 2 (P2) is a male infant born prematurely at week 36 via cesarean section, weighing 2.6 kg at birth. The patient was initially well but developed watery diarrhea at two weeks of life, requiring multiple admissions to local hospitals. Family history is notable for a consanguineous marriage. Previous hospital records reveal that he had severe anemia requiring blood transfusions, and several formula changes were made, but the patient did not show any improvement. He was maintained on Total Parenteral Nutrition (TPN) due to persistent diarrhea and failure to thrive.

On admission to our facility at 41 days of life, he was malnourished, weighing 2.4 kg (Z-score: −4.3), and suffered multiple sepsis episodes, requiring various NICU admissions. He was maintained on TPN and given multiple albumin transfusions. Comprehensive immunodeficiency workup was negative. Moreover, endoscopy with histopathology evaluation was normal.

By 5 months of age, he tolerated limited enteral feeding with elemental formula and a low-fat diet, allowing discontinuation of TPN. WES identified a mutation in *DGAT1* gene.

The patient was discharged after 118 days of hospitalization, hemodynamically stable, and his weight improved to 5.5 kg (Z-score: −2.7). He continues to follow up and gain weight. At 196 days of life, he weighed 6.7 kg (Z-score: −1.6) and advancing his diet without diarrhea recurrence, as in Figure 1b.

Patient 3 (P3) is a full-term female infant (birthweight 2.7 kg) born to consanguineous parents. She was healthy until 7 months of age, when she developed recurrent vomiting and foul-smelling diarrhea associated with generalized edema. On admission to our facility, her weight was 5.7 kg (Z-score: −2.8). Differential diagnoses included immunodeficiency and abetalipoproteinemia, but endoscopic biopsies and immunologic panels were unremarkable.

Nutritional interventions with a hydrolyzed formula were ineffective, whereas an amino acid–based low-fat formula supplemented with MCT oil and fat-soluble vitamins led to clinical stabilization. She additionally received albumin infusions for protein-losing enteropathy and IVIG for hypogammaglobulinemia. With this approach, her diarrhea resolved, and she gradually gained weight by 9 months of age.

WES revealed a novel homozygous missense mutation in *DGAT1*, consistent with the clinical phenotype of congenital diarrhea and protein-losing enteropathy. At her most recent follow-up, she remained clinically stable on incremental dietary advances, normalization of serum albumin levels, and no recurrence of diarrhea or edema. Her growth trajectory is depicted in Figure 1c.

All three patients presented with the characteristic Diarrhea 7, Protein-Losing Enteropathy Type (DIAR7) phenotype. Additional features included coagulopathies secondary to vitamin K deficiency in Patient 1 and 3. Serum triglycerides were elevated in Patients 1 and 2, whereas Patient 3 also presented with normal triglyceride levels.

### Genetic analysis and findings

Genomic DNA was extracted from peripheral blood leukocytes of the probands and available family members. WES was performed using the IDT xGen Exome Research Panel (v1.0/v2.0) (Integrated DNA Technologies, Coralville, IA, USA) with paired-end (2 × 150 bp) sequencing on Illumina NovaSeq 6000 Platform (Illumina, San Diego, CA, USA). Sequence data were processed using an in-house EMQN-certified pipeline with alignment to the human reference genome (hg19) using the DRAGEN pipeline (DRAGEN Bio-IT Platform, Illumina, San Diego, CA, USA) on DNAnexus (DNAnexus, Mountain View, CA, USA). The mean on-target coverage ranged from 73× to 167×, with >95% of target regions covered at ≥20× in one sample. Variant filtering excluded common variants (minor allele frequency >0.1% in gnomAD), intronic variants beyond ±6 bp of splice sites, and synonymous variants beyond ±3 bp unless predicted to affect splicing.

WES of the three patients revealed homozygous variants in the DGAT1 gene. For patient 1, WES identified a homozygous *DGAT1* variant (NC_000008.11: g. 144317087G>A; NM_012079.6: c.1183C>T; p.Arg395Ter). The variant has an extremely low frequency in the gnomAD v4.1 (AF = 4.84 × 10^−5^) in 1,612,844 control chromosomes, with no homozygous occurrence. It is a nonsense variant, predicted to result in loss or disruption of normal protein function through nonsense-mediated decay (NMD). Multiple pathogenic variants have been reported downstream of this position. In ClinVar, this variant has been classified as likely pathogenic by five independent submitters (ClinVar ID: VCV001685693.7).

The WES results for patient 2 identified the homozygous variant (NC_000008.11: g. 144317713C>T; NM_012079.6: c.895-1G>A), a canonical splice acceptor site mutation that abolishes the intron 10/exon 11 boundary and causes aberrant splicing. The allele frequency of this variant in the gnomAD v4.1 database is (AF = 1.86 × 10^−6^) in 1,461,462 control chromosomes, with no homozygous occurrence. This variant has been previously documented in affected individuals and is classified as pathogenic (3–5), demonstrating a consistent genotype-phenotype association.

**Figure 2:**
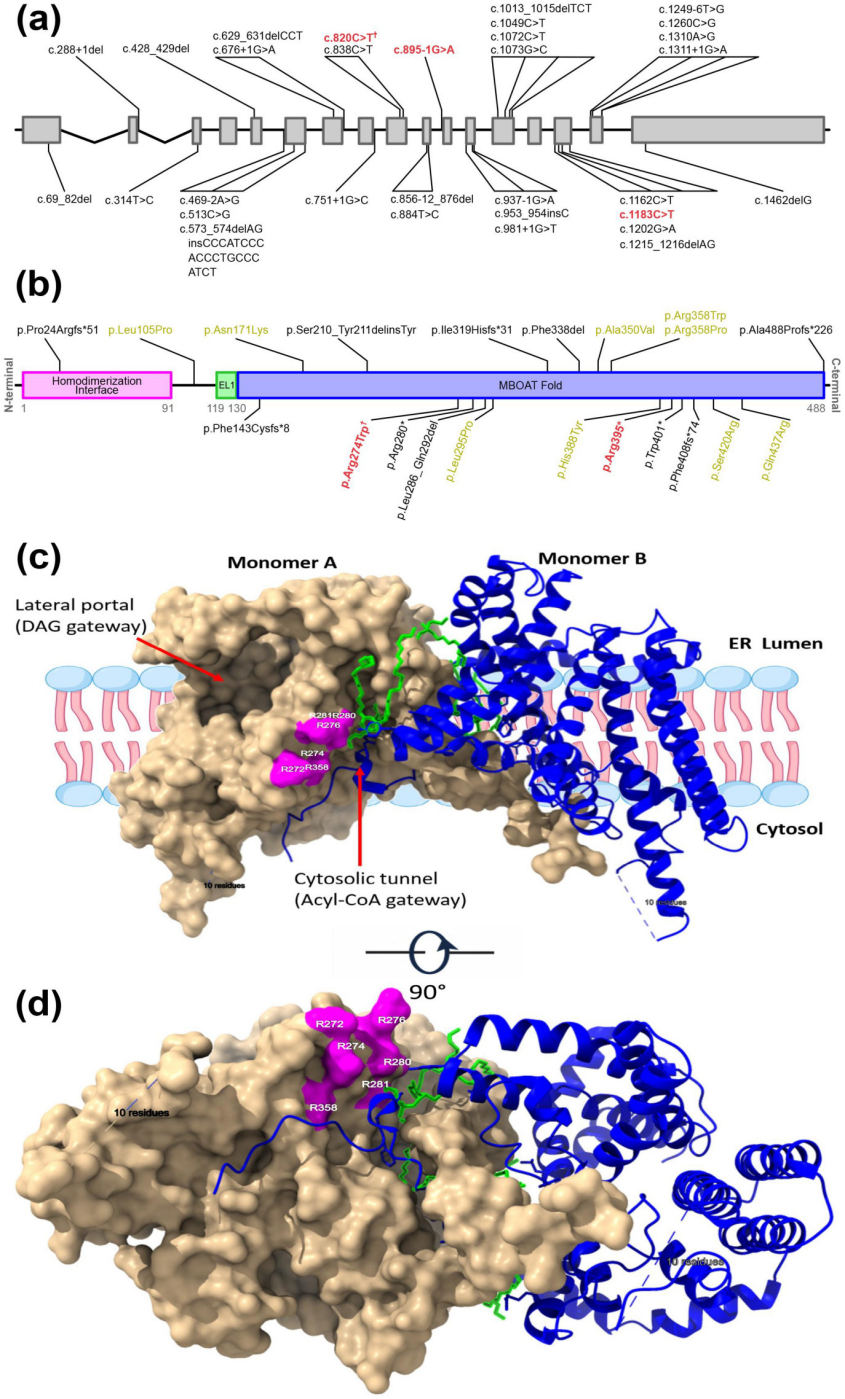
DGAT1 gene structure and enzyme architecture. Schematic representation of the DGAT1 gene with solid boxes representing exons and intronic regions as connecting lines **(a)**. The variants labelled in black have been previously reported as pathogenic in the literature, with variants identified in this study highlighted in red. Introns between exon 1,2 and 3 have been shortened to conserve space. † annotates the novel variant. **(b)** Two-dimensional protein domain map showing the three main domains and the positions of the variants corresponding to the mutations in panel **(a)**. The two coding variants identified in our study are shown in red. The splice-site variant c.895-1G>A is not displayed on the protein map because it does not correspond to a translated amino acid residue. Variants in yellow are missense variants, whereas variants in black represent truncating, splice-site, in-frame deletions, or complex variants. Missense variants appear to cluster within the MBOAT domain, with the exception of p.Leu105Pro, whereas non-missense variants show no clear clustering. **(c,d)** The enzyme forms a homodimer in the endoplasmic reticulum membrane, with one protomer shown as a light-beige surface and the other as a dark-blue TM helical cartoon. Acyl-CoA enters from the cytosolic side, while diacylglycerol (DAG) accesses the active site through a lateral membrane portal, with both substrates converging in a central reaction chamber. The magenta-highlighted residues (Arg272, Arg274, Arg276, Arg280, Arg281, and Arg358) form a positively charged arginine cluster likely involved in acyl-CoA positioning (shown in green). **(c)** is a side view, while **(d)** is a bottom view. The model was generated using UCSF ChimeraX based on the cryo-EM–solved dimeric structure of the protein in complex with 2 mM oleoyl-CoA (PDB accession number: 6VYI).

Interestingly, WES analysis of patient 3 identified a *DGAT1* missense variant (Figure 2a), (NC_000008.11: g.144317949G > A; NM_012079.6: c.820C > T; p.Arg274Trp). This variant is present in gnomAD v4.1 with a very low allele frequency (AF = 1.78 × 10^−5^) in 1,519,434 control chromosomes, with no homozygous occurrence. ClinVar lists two submissions with conflicting pathogenicity classifications. To clarify its relevance in this family, we performed Sanger sequencing, which demonstrated perfect segregation with the clinical phenotype, consistent with an autosomal recessive inheritance pattern (Figure 1d). University of California, Santa Cruz (UCSC) 100-vertebrate conservation analysis shows that Arg274 is nearly completely conserved across vertebrates, from higher apes to fishes, with a high phyloP 100 score of 5.556 (6). This strong constraint value supports the functional importance of Arg274 and suggests that substitutions at this position are likely deleterious. Consistently, the majority of *in silico* prediction tools from the GeneBe server classify this missense variant as pathogenic or deleterious (https://genebe.net/).

To strengthen variant interpretation, we formally classified all three DGAT1 variants according to the ACMG/AMP guidelines and listed the relevant criteria for each variant in Table 1. The two truncating/splice-disrupting variants (patients 1 and 2) were classified as pathogenic, whereas the missense variant p.Arg274Trp for patient 3 was classified as likely pathogenic. The relevant *in silico* prediction scores were also added, including CADD (7), REVEL (8), MetaRNN (9), and AlphaMissense (10) for the missense variant, and SpliceAI (11), Pangolin (12), and dbscSNV (13) for the splice-site variant.

**Table 1:** Formal ACMG/AMP classification, applied evidence criteria, and in silico predictions for the three DGAT1 variants identified in this study.

Patient	Variant	ACMG/AMP classification	Applied criteria	*In silico* prediction
1	NM_012079.6(DGAT1):c.1183C>T(p.Arg395*)	Pathogenic	PVS1, PP1, PM2, PP5_Very_Strong	CADD: 41
2	NM_012079.6:c.895-1G>A (Acceptor splice loss)	Pathogenic	PVS1, PP1-S, PM2	CADD: 35 SpliceAI: 0.99 Pangolin: 0.77 dbscSNV1_RF: 0.94
3	NM_012079.6(DGAT1):c.820C > T (p.Arg274Trp)	Likely Pathogenic	PP1-S, PM2, PP3_Strong	CADD: 33 REVEL: 0.799 MetaRNN: 0.97 AlphaMissense: 0.93

In silico annotations and prediction scores were retrieved from the GeneBe platform and Ensembl Variant Effect Predictor (VEP). Splicing impact for NM_012079.6:c.895-1G > A was additionally evaluated using SpliceAI Lookup, Pangolin, and dbscSNV.

### In silico structural analysis of p.Arg274Trp

To assess pathogenicity, we examined the structural and mechanistic context of Arg274 within DGAT1. DGAT1 is a multi-pass endoplasmic reticulum (ER) membrane homodimer enzyme. Each monomer is composed of nine transmembrane (TM) helices. Two different high-resolution cryo-EM 3D models revealed that each substrate enters through a distinct passageway: Diacylglycerol (DAG) enters laterally from the membrane via a passageway formed by TM4 and TM6, and facing the hydrophobic core of the ER membrane, while acyl-Coenzyme A (CoA) approaches from the cytosolic side through a dedicated tunnel (14, 15) (Figures 2c,d). Modeling of the Arg274Trp substitution using UCSF ChimeraX rotamer selection, as well as analysis with the Missense3D server (16), did not show an obvious direct effect on local or global protein stability. However, examination of Arg274 in the context of its position strongly supports its functional importance. The Arg274 residue is located at the cytosolic end of TM5, facing the cytosolic portal that forms the entry site for the acyl-CoA substrate. This residue stabilizes the TM5 at the membrane interface. Several lines of evidence support a functionally disruptive effect of the Arg274Trp missense variant. The substitution replaces the positively charged Arginine with Tryptophan, a bulky hydrophobic aromatic residue, at the ER membrane-cytosol interface. This change is predicted to disrupt the IL1–TM5 hinge, altering TM5 packing and tilt, thereby distorting the geometry of acyl-CoA access via the cytosolic portal. This interpretation is further supported by the pathogenic TM5 variant DGAT1: p.Leu295Pro reported in the ClinVar database (Accession: VCV000217459.4), demonstrating that TM5 is functionally sensitive to structural perturbation. In addition, Arg274 lies within a conserved cluster of positively charged di-arginine motifs (RXR/RXXR), localized at the cytosolic tips of TM4 and TM5, immediately adjacent to the acyl-CoA cytosolic entry portal (Figures 2c,d). These positively charged motifs are thought to play a critical role in membrane trafficking and substrate positioning as acyl-CoA enters through its Coenzyme A (CoA) headgroup, which is highly hydrophilic and negatively charged (17). Molecular dynamics simulations also show that positively charged residues on the cytosolic face guide acyl-CoA toward the active site, underscoring the importance of maintaining a proper electrostatic environment (18).

## Discussion

Biallelic variants in DGAT1 are an emerging cause of CODEs, presenting in early infancy with intractable diarrhea, protein-losing enteropathy, and failure to thrive (19). The first affected individuals were reported in 2012, when functional studies confirmed loss of DGAT1 activity leading to the DIAR7 phenotype (19). A structured literature search was conducted using PubMed/MEDLINE, Embase, Scopus and Google Scholar with keywords including “DGAT1”, “DGAT1 deficiency”, “DGAT1 mutation” combined with phenotypic terms such as: “congenital diarrhea”, “protein-losing enteropathy” and “failure to thrive”. Only genetically confirmed cases were included. To date, 39 patients with 29 unique mutations have been described (Supplementary Table S1). Our report adds three additional cases, including one novel variant, to better highlight the genotype-phenotype correlations. A summary of mutation types and associated clinical trends is provided in Table 2.

**Table 2:** Summary of DGAT1 mutation types and associated clinical trends based on reported cases in the literature.

Mutation type	Frequency (%)	Average age of onset	Clinical severity	Key features
Truncating	17 (40.5%)	34 days	Severe	Early onset; severe diarrhea and PLE; marked hypoalbuminemia; frequent infections; TPN needed; higher mortality
Splice-site	8 (19.0%)	63 days	Moderate	loss-of-function phenotype; hypoalbuminemia; infections common; variable severity
Missense	6 (14.3%)	73 days	Mild	Later onset; milder course; suggests residual function; lower mortality
In-frame deletion/complex variant	11 (26.2%)	28 days	Moderate	Early onset; intermediate severity; variable phenotype; better outcomes than truncating variants

Mutation categories are derived from genetically confirmed DGAT1 deficiency cases compiled from the literature (Supplementary Table S1). Mean age of onset is presented for each mutation group. For splice-site variants, the mean was calculated after exclusion of a single outlier case with markedly delayed presentation (30 months), to better reflect the typical early-onset phenotype. Clinical severity was qualitatively assessed based on reported outcomes and clinical parameters, including hypoalbuminemia, presence of infections, and survival status, and categorized as severe, moderate, or mild to reflect overall disease burden across mutation types.

PLE, protein-losing enteropathy; TPN, total parenteral nutrition.

Loss of DGAT1 function leads to impaired chylomicron assembly, failure of lipid droplet formation, and lipotoxicity in enterocytes resulting in polarity defects and malabsorption (20, 21). Although some patients demonstrate villous atrophy, many, including our cases, have normal histology; therefore, normal endoscopic and histopathologic findings should not exclude DGAT1 deficiency in the appropriate clinical context. Affected individuals typically develop watery diarrhea within days to weeks of life; however, later presentations have been described (3, 4, 22), and reported ages at onset in the literature range from the neonatal period to 30 months. Late presentations have been described, particularly in individuals with milder missense changes (3). Our third case, presenting at 7 months of age with a homozygous missense variant, further supports the possibility that some missense variants retain partial residual activity and may be associated with delayed or milder phenotypes. This aligns with the broader phenotypic spectrum and underscores that DGAT1-related disease should remain a diagnostic consideration beyond the neonatal period.

Within the broader spectrum of monogenic congenital diarrheal disorders, DGAT1 deficiency has several distinguishing features. Unlike disorders primarily caused by epithelial transport defects, such as congenital chloride or sodium diarrhea, DGAT1 deficiency results from impaired intracellular lipid metabolism and enterocyte lipotoxicity. Histology may be normal or only mildly abnormal, in contrast to disorders with prominent villous atrophy or epithelial architectural abnormalities. Biochemically, the combination of hypoalbuminemia, protein-losing enteropathy, hypogammaglobulinemia, and frequently hypertriglyceridemia may provide an important diagnostic clue. Clinically, the marked response to a low-fat, amino acid-based diet further helps distinguish DGAT1 deficiency from other early-onset enteropathies in which nutritional modification alone is less specifically effective.

Diarrhea and its sequelae, such as metabolic acidosis, electrolyte disturbances, steatorrhea, and fat-soluble vitamin deficiencies, are often the first presenting symptom (4, 21, 23, 24). Hypoalbuminemia and hypogammaglobulinemia are common, and opportunistic infections may occur, usually associated with central venous catheters for TPN (4, 20, 25, 26). Notably, all three patients developed infections, including central line–associated bloodstream infections (CLABSI), highlighting the vulnerability of patients with DGAT1 deficiency, whereas infections have been reported less consistently in the prior literature (20). Hypertriglyceridemia is a paradoxical but commonly reported feature which may reflect disrupted intestinal handling, altered chylomicron assembly, and compensatory systemic lipid metabolic changes, particularly in the setting of intestinal failure and TPN administration (21, 23). Several patients, including those in our series, demonstrated prolonged international normalized ratio (INR) and activated partial thromboplastin time (aPTT) values likely reflecting vitamin K deficiency from fat malabsorption. Prognosis varies widely, with severe loss-of-function mutations linked to poorer outcomes (19, 23).

Nutritional therapy remains central. Our patients improved with amino acid–based low-fat formulas supplemented with MCT oil and essential fatty acids, consistent with recent cohort data (5). Hydrolyzed or high-MCT formulas were ineffective, echoing published recommendations (5, 27). Supportive measures included albumin replacement, fat-soluble vitamin supplementation, and IVIG in patients with hypogammaglobulinemia. Regular monitoring of growth, electrolytes, triglycerides, and, ideally, fecal *α*1-antitrypsin (not performed in our series) is advised (5, 26).

This case series expands the genotypic and phenotypic spectrum of DGAT1-related congenital diarrhea and protein-losing enteropathy. Our findings emphasize the importance of considering DGAT1 deficiency in neonates presenting with intractable diarrhea, hypoalbuminemia, and failure to thrive, particularly in populations with a high prevalence of consanguinity. Nutritional management with low-fat, amino acid–based formulas, along with supportive care, can significantly improve growth and clinical outcomes.

## Data Availability

The datasets presented in this study can be found in online repositories. The names of the repository/repositories and accession number(s) can be found in the article/Supplementary Material.

## References

[1] Kijmassuwan T Balouch F. Approach to congenital diarrhea and enteropathies (CODEs). Indian J Pediatr. (2024) 91(6):598–605. 10.1007/s12098-023-04929-7 38105403

[2] McFie PJ Stone SL Banman SL Stone SJ. Topological orientation of acyl-CoA:diacylglycerol acyltransferase-1 (DGAT1) and identification of a putative active site histidine and the role of the N terminus in dimer/tetramer formation. J Biol Chem. (2010) 285(48):37377–87. 10.1074/jbc.M110.163691 20876538 PMC2988343

[3] Ye Z Huang Y Wang Y Lu J Wu J Yu Z. Phenotype and genotype of a cohort of Chinese children with early-onset protein-losing enteropathy. J Pediatr. (2019) 208:38–42.e3. 10.1016/j.jpeds.2018.12.003 30853196

[4] Xu L Gu W Luo Y Lou J Chen J. DGAT1 Mutations leading to delayed chronic diarrhoea: a case report. BMC Med Genet. (2020) 21:239. 10.1186/s12881-020-01164-1 33261563 PMC7708908

[5] Zheng Y Li Y Zheng C Yang L Zhang C Huang Y A low-fat amino acid diet reverses intestinal failure and shows good growth trends in five infants with diacylglycerol transferase 1 (DGAT1) deficiency: a prospective cohort study. Lipids Health Dis. (2024) 23(1):379. 10.1186/s12944-024-02348-x 39548446 PMC11566179

[6] Lee CM Barber GP Casper J Clawson H Diekhans M Gonzalez JN UCSC Genome browser enters 20th year. Nucleic Acids Res. (2020) 48(D1):D756–61. 10.1093/nar/gkz1012 31691824 PMC7145642

[7] Schubach M Maass T Nazaretyan L Röner S Kircher M. CADD V1.7: using protein language models, regulatory CNNs and other nucleotide-level scores to improve genome-wide variant predictions. Nucleic Acids Res. (2024) 52(D1):D1143–54. 10.1093/nar/gkad989 38183205 PMC10767851

[8] Ioannidis NM Rothstein JH Pejaver V Middha S McDonnell SK Baheti S REVEL: an ensemble method for predicting the pathogenicity of rare missense variants. Am J Hum Genet. (2016) 99(4):877–85. 10.1016/j.ajhg.2016.08.016 27666373 PMC5065685

[9] Li C Zhi D Wang K Liu X. MetaRNN: differentiating rare pathogenic and rare benign missense SNVs and InDels using deep learning. Genome Med. (2022) 14(1):115. 10.1186/s13073-022-01120-z 36209109 PMC9548151

[10] Tordai H Torres O Csepi M Padányi R Lukács GL Hegedűs T. Analysis of AlphaMissense data in different protein groups and structural context. Sci Data. (2024) 11(1):495. 10.1038/s41597-024-03327-8 38744964 PMC11094042

[11] Jaganathan K Panagiotopoulou SK McRae JF Darbandi SF Knowles D Li YI Predicting Splicing from Primary Sequence with Deep Learning [Internet]. Available online at: https://www.cell.com/cell/abstract/S0092-8674(18)31629-5 (Accessed April 13, 2026). 10.1016/j.cell.2018.12.015 30661751

[12] Zeng T Li YI. Predicting RNA splicing from DNA sequence using pangolin. Genome Biol. (2022) 23(1):103. 10.1186/s13059-022-02664-4 35449021 PMC9022248

[13] Jian X Boerwinkle E Liu X. In silico prediction of splice-altering single nucleotide variants in the human genome. Nucleic Acids Res. (2014) 42(22):13534–44. 10.1093/nar/gku1206 25416802 PMC4267638

[14] Wang L Qian H Nian Y Han Y Ren Z Zhang H Structure and mechanism of human diacylglycerol O-acyltransferase-1. Nature. (2020) 581(7808):329. 10.1038/s41586-020-2280-2 32433610 PMC7255049

[15] Sui X Wang K Gluchowski NL Elliott SD Liao M Walther TC Structure and catalytic mechanism of a human triglyceride synthesis enzyme. Nature. (2020) 581(7808):323. 10.1038/s41586-020-2289-6 32433611 PMC7398557

[16] Ittisoponpisan S Islam SA Khanna T Alhuzimi E David A Sternberg MJE. Can predicted protein 3D structures provide reliable insights into whether missense variants are disease associated? J Mol Biol. (2019) 431(11):2197–212. 10.1016/j.jmb.2019.04.009 30995449 PMC6544567

[17] Winichayakul S Xue H Roberts N. A conserved N-terminal di-arginine motif stabilizes plant DGAT1 and modulates lipid droplet organization. Int J Mol Sci. (2025) 26(15):7406. 10.3390/ijms26157406 40806535 PMC12347784

[18] Lee H Im W. Substrates (acyl-CoA and diacylglycerol) entry and products (CoA and triacylglycerol) egress pathways in DGAT1. J Comput Chem. (2025) 46(11):e70108. 10.1002/jcc.70108 40251888 PMC12008735

[19] Haas JT Winter HS Lim E Kirby A Blumenstiel B DeFelice M DGAT1 Mutation is linked to a congenital diarrheal disorder. J Clin Invest. (2012) 122(12):4680. 10.1172/JCI64873 23114594 PMC3533555

[20] van Rijn JM Ardy RC Kuloğlu Z Härter B van Haaften-Visser DY van der Doef HP Intestinal failure and aberrant lipid metabolism in patients with DGAT1 deficiency. Gastroenterology. (2018) 155(1):130. 10.1053/j.gastro.2018.03.040 29604290 PMC6058035

[21] Schlegel C Lapierre LA Weis VG Williams JA Kaji I Pinzon-Guzman C Reversible deficits in apical transporter trafficking associated with DGAT1 deficiency. Traffic. (2018) 19(11):879. 10.1111/tra.12608 30095213 PMC6191315

[22] Valentini MA Fedrizzi VA Krochik AG Abbate S Ferrari M Contreras MB DGAT1 Mutation in two sisters with failure to thrive: a case report. Arch Argent Pediatr. (2023) 121(1):e202202606. 10.5546/aap.2022-02606.eng 36315449

[23] Eldredge JA Couper MR Barnett CP Rawlings L Couper RTL. New pathogenic mutations associated with diacylglycerol O-acyltransferase 1 deficiency. J Pediatr. (2021) 233:268–72. 10.1016/j.jpeds.2021.02.028 33607125

[24] Li J Sun M Guo J Xu L. Case report: diagnosis and treatment of DGAT1 deficiency-induced congenital diarrhea in two cases and literature review. Front Pediatr. (2023) 11:1253800. 10.3389/fped.2023.1253800 37908965 PMC10613706

[25] Stephen J Vilboux T Haberman Y Pri-Chen H Pode-Shakked B Mazaheri S Congenital protein losing enteropathy: an inborn error of lipid metabolism due to DGAT1 mutations. Eur J Hum Genet. (2016) 24(9):1268. 10.1038/ejhg.2016.5 26883093 PMC4989215

[26] Ratchford TL Kirby AJ Pinz H Patel DR. Congenital diarrhea from DGAT1 mutation leading to electrolyte derangements, protein-losing enteropathy, and rickets. J Pediatr Gastroenterol Nutr. (2018) 66(3):e82–3. 10.1097/MPG.0000000000001750 28937539

[27] Millman P Rimon RM Toff C Engvall M Shaoul R Wilschanski M A novel nutritional approach to infants and children with congenital diarrhea due to homozygous DGAT1 mutations. J Pediatr Gastroenterol Nutr. (2024) 79(2):250–8. 10.1002/jpn3.12241 38934410

