# Supplementary material for: Case Report: DGAT1 deficiency in three infants including a novel missense variant: structural insights and comparison with reported cases
Source: Front Pediatr. 2026 May 12;14:1829390. doi: 10.3389/fped.2026.1829390 (PMC13201459; doi:10.3389/fped.2026.1829390)
Supplement: Supplementary file 1 [file Table1.docx]

**Table S1**

*Clinical and genetic characteristics of patients with DGAT1 deficiency reported in the literature*

| **Reference** | **Patient** | **Sex** | **Descent** | **Consanguinity** | **DGAT1 mutation site** | **Protein Variant** | **Mode of Inheritance** | **Onset** | **Serum TG** | **γ-globulin** | **Infections** | **Albumin** | **A1AT** | **Clinical outcome** |
| --- | --- | --- | --- | --- | --- | --- | --- | --- | --- | --- | --- | --- | --- | --- |
| Haas et al. | 1 | F | Ashkenazi Jewish | – | g.13827T>C | p.Ala226_Arg250del | homozygous | 3 Days | H | L | + | L | H | Died |
|  | 2 | M | Ashkenazi Jewish | – | g.13827T>C | p.Ala226_Arg250del | homozygous | 3 Days | H | N | NA | L | H | Alive |
| Stephen et al. | 3 | M | Arab | + | c.884T>C | p.Leu295Pro | homozygous | 2 months | H | L | NA | L | H | Alive |
|  | 4 | M | Ashkenazi Jewish | – | g.13827T>C | p.Ala226_Arg250del | homozygous | 8 days | N | N | NA | L | H | Alive |
|  | 5 | M | Ashkenazi Jewish | – | g.13827T>C | p.Ala226_Arg250del | homozygous | After birth | H | N | + | L | H | Alive |
| Gluchwoski et al. | 6 | M | South Asian | NA | c.314C>T | p.Leu105Pro | homozygous | After birth | H | L | NA | L | H | Alive |
|  | 7 | M | South Asian | NA | c.314C>T | p.Leu105Pro | homozygous | After birth | NA | NA | NA | NA | H | Alive |
| Ratchford et al. | 8 | F | Caucasian | NA | c.1013_1015delTCT (maternal) | p.Phe338del | Compound heterozygous | 1 month | N | L | NA | L | H | Alive |
|  |  |  |  |  | c.1260C>G (paternal) | p.Ser420Arg |  |  |  |  |  |  |  |  |
| Rijn et al. | 9 | M | Turkish | + | c.1202G>A | p.Trp401* | homozygous | After birth | NA | L | + | L | NA | Alive |
|  | 10 | F | Turkish | + | c.1202G>A | p.Trp401* | homozygous | After birth | NA | L | + | L | NA | Died |
|  | 11 | F | Turkish | + | c.573_574delAGinsCCCATCCCACCCTGCCCATCT | p. ? | homozygous | 3 weeks | NA | L | NA | L | N | Alive |
|  | 12 | M | Turkish | + | c.937-1G>A | p. ? | homozygous | 2 months | NA | L | NA | L | NA | Alive |
|  | 13 | M | Turkish | + | c.953insC | p.Ile319Hisfs*31 | homozygous | 40 days | NA | L | NA | L | NA | Alive |
|  | 14 | M | Turkish | + | c.953insC | p.Ile319Hisfs*31 | homozygous | 2.5 months | NA | L | + | L | NA | Alive |
|  | 15 | M | Dutch Caucasian | + | c.629_631delCCT | p.Ser210_Tyr211delinsTyr | homozygous | 1 month | NA | N | NA | N | NA | Alive |
|  | 16 | M | Dutch Caucasian | + | c.629_631delCCT | p.Ser210_Tyr211delinsTyr | homozygous | 1 month | NA | N | NA | N | NA | Alive |
|  | 17 | F | Dutch Caucasian | NA | c.629_631delCCT | p.Ser210_Tyr211delinsTyr | homozygous | After birth | NA | L | + | L | H | Alive |
|  | 18 | F | Dutch Caucasian | NA | c.629_631delCCT | p.Ser210_Tyr211delinsTyr | homozygous | After birth | NA | L | + | L | H | Alive |
| Schlegel et al. | 19 | M | Hispanic | NA | g.13827T>C | p.Ala226_Arg250del | homozygous | 7 weeks | NA | L | + | L | N | Alive |
| Ye et al. | 20 | F | Han & Uyghur Chinese | – | c.895-1G>A | p. ? | homozygous | After birth | NA | L | NA | L | NA | Died |
|  | 21 | F | Han & Uyghur Chinese | – | c.1249-6T>G | p. ? | homozygous | 30 months | NA | L | NA | L | NA | Died |
| Gupta et al. | 22 | M | NA | + | c.629_631delCCT | p.Ser210del | homozygous | 2 weeks | NA | NA | NA | NA | NA | Alive |
|  | 23 | F | NA | NA | c.1310A>G | p.Gln437Arg | Phase Unknown | 3 weeks | NA | NA | NA | NA | NA | NA |
|  |  |  |  |  | c.981+1G>T (maternal) | p. ? |  |  |  |  |  |  |  |  |
|  | 24 | F | Mexican | + | c.676+1G>A | p. ? | homozygous | 11 days | NA | NA | NA | NA | NA | Alive |
|  | 25 | F | NA | NA | c.1311+1G>A | p. ? | Phase Unknown | 1 week | NA | L | NA | N | NA | Alive |
|  |  |  |  |  | c.1462delG | p.Ala488Profs*226 |  |  |  |  |  |  |  |  |
| Xu et al. | 26 | F | Chinese | – | c.895-1G>A (paternal) | p. ? | Compound heterozygous | 8 months | H | L | + | L | NA | Alive |
|  |  |  |  |  | c.751+1G>C (maternal) | p. ? |  |  |  |  |  |  |  |  |
| Eldredge et al. | 27 | M | NA | NA | c.288+1del | p. ? | Compound heterozygous | 17 days | NA | L | + | L | H | Alive |
|  |  |  |  |  | c.629_631del | p.Ser210del |  |  |  |  |  |  |  |  |
|  | 28 | F | Caucasian | – | c.428_429del | p.Phe143Cysfs*8 | Compound heterozygous | After birth | NA | NA | + | L | H | Alive |
|  |  |  |  |  | c.629_631del (paternal) | p.Ser210del |  |  |  |  |  |  |  |  |
| Bijker et al. | 29 | F | Turkish | + | c.469-2A>G | p. ? | homozygous | 2.5 months | NA | L | + | L | NA | Alive |
| Li et al. | 30 | F | Chinese | NA | c.1215_1216delAG | p.Phe408fs*74 | Compound heterozygous | 3 months | NA | L | NA | L | NA | NA |
|  |  |  |  |  | c.838C>T | p.Arg280* |  |  |  |  |  |  |  |  |
|  | 31 | M | Chinese | NA | c.1049C>T | p.Ala350Val | Compound heterozygous | After birth | H | L | NA | L | NA | NA |
|  |  |  |  |  | c.1215_1216delAG | p.Phe408fs*74 |  |  |  |  |  |  |  |  |
| Valentini et al. | 32 | F | Latin American | NA | c.1162C>T | p.His388Tyr | Compound heterozygous | 4 months | NA | L | NA | L | H | Alive |
|  |  |  |  |  | c.838C>T | p.Arg280* |  |  |  |  |  |  |  |  |
|  | 33 | F | Latin American | NA | c.1162C>T | p.His388Tyr | Compound heterozygous | 2 months | NA | L | NA | L | N | Alive |
|  |  |  |  |  | c.838C>T | p.Arg280* |  |  |  |  |  |  |  |  |
| Shi et al. | 34 | F | Chinese | – | c.1215_1216delAG (paternal) | p.Arg280* | Compound heterozygous | 2 days | H | L | NA | L | NA | Alive |
|  |  |  |  |  | c.838C>T (maternal) | p.Phe408fs*74 |  |  |  |  |  |  |  |  |
| Zheng et al. | 35 | M | Han Chinese | – | c.1073G>C | p.Arg358Pro | Compound heterozygous | 3 days | H | NA | NA | L | NA | Alive |
|  |  |  |  |  | c.1072C>T | p.Arg358Trp |  |  |  |  |  |  |  |  |
|  | 36 | F | Han Chinese | – | c.856-12_876del | p.Leu286_Gln292del | Compound heterozygous | 14 days | N | NA | NA | L | NA | Alive |
|  |  |  |  |  | c.69_82del | p.Pro24Argfs*51 |  |  |  |  |  |  |  |  |
|  | 37 | F | Han Chinese | – | c.1215_1216delAG | p.Phe408fs*74 | Compound heterozygous | 3 days | H | NA | NA | L | NA | Alive |
|  |  |  |  |  | c.838C>T | p.Arg280* |  |  |  |  |  |  |  |  |
|  | 38 | M | Han Chinese | – | c.1215_1216delAG | p.Phe408fs*74 | Compound heterozygous | 3 days | H | NA | NA | L | NA | Alive |
|  |  |  |  |  | c.1049C>T | p.Ala350Val |  |  |  |  |  |  |  |  |
|  | 39 | F | Han Chinese | – | c.895-1G>A | p. ? | Compound heterozygous | 12 days | H | NA | NA | L | NA | Alive |
|  |  |  |  |  | c.513C>G | p.Asn171Lys |  |  |  |  |  |  |  |  |
| **This Case** | **40** | **M** | **Arab** | **+** | **c.1183C>T** | **p.Arg395*** | **homozygous** | **10 days** | **H** | **L** | **+** | **L** | **NA** | **NA** |
|  | **41** | **M** | **Arab** | **+** | **c.895-1G>A** | **p. ?** | **homozygous** | **14 days** | **H** | **L** | **+** | **L** | **NA** | **Alive** |
|  | **42** | **F** | **Arab** | **+** | **c.820C>T** | **p.Arg274Trp** | **homozygous** | **6 months** | **H** | **L** | **+** | **L** | **NA** | **Alive** |

*Note*: *This table summarizes both genetic and clinical features of all patients reported in the literature to have DGAT1 mutations. Abbreviations: F, female; M, male; Serum TG, serum triglycerides; γ-globulin, serum gamma globulins; A1AT, fecal alpha-1 antitrypsin; H, high; L, low; N, normal; +, present; –, absent; NA, not applicable or has not been measured in the study referenced.*
